# Supplementary material for: Natural and laboratory mutations in kuzbanian are associated with zinc stress phenotypes in Drosophila melanogaster
Source: Sci Rep. 2017 Feb 20;7:42663. doi: 10.1038/srep42663 (PMC5316978; doi:10.1038/srep42663)

Supplementary Information File

Natural and laboratory mutations in *kuzbanian* are associated with zinc stress phenotypes in *Drosophila melanogaster*

Hung Le Manh[1,2], Lain Guio[1], Miriam Merenciano[1], Quirze Rovira[1], Maite G. Barrón[1] and Josefa González[1*]

Table S1. Fly stocks in which the presence, absence or heterozygous state of *FBti0019170* has been determined.

No	Strains	PCR's results			Genotype	Origin
		Present	Absent	Heterozygous		
1	#5816		X		w[*]; P{w[+mC]=UAS-kuz.F}DF1	Bloomington
2	#4414	X			y[1] w[*]; P{w[+mC]=Act5C-GAL4}25FO1/CyO, y[+]	Bloomington
3	#7087		X		w[*]; lin[G2]/CyO	Bloomington
4	B1		X		wild-type strain	Sweden (SW)
5	B2		X		wild-type strain	SW
6	B4		X		wild-type strain	SW
7	B5		X		wild-type strain	SW
8	B6			X	wild-type strain	SW
9	B7	X			wild-type strain	SW
10	B8		X		wild-type strain	SW
11	B9			X	wild-type strain	SW
12	B10			X	wild-type strain	SW
13	B11		X		wild-type strain	SW
14	B12		X		wild-type strain	SW
15	B13			X	wild-type strain	SW
16	B14		X		wild-type strain	SW
17	B15		X		wild-type strain	SW
18	B17			X	wild-type strain	SW
19	B18		X		wild-type strain	SW
20	B19			X	wild-type strain	SW
21	B21			X	wild-type strain	SW
22	B22			X	wild-type strain	SW
23	B24			X	wild-type strain	SW
24	B25			X	wild-type strain	SW
25	B26		X		wild-type strain	SW
26	B27		X		wild-type strain	SW
27	B28		X		wild-type strain	SW
28	B29			X	wild-type strain	SW
29	B30			X	wild-type strain	SW
30	B31		X		wild-type strain	SW
31	B33		X		wild-type strain	SW
32	B35			X	wild-type strain	SW
33	B36		X		wild-type strain	SW
34	B38			X	wild-type strain	SW
35	B39		X		wild-type strain	SW
36	B42			X	wild-type strain	SW

37	B44		X		wild-type strain	SW
38	B45	X			wild-type strain	SW
39	B46			X	wild-type strain	SW
40	B47		X		wild-type strain	SW
41	B48		X		wild-type strain	SW
42	S1		X		wild-type strain	SW
43	S2		X		wild-type strain	SW
44	S3		X		wild-type strain	SW
45	S4		X		wild-type strain	SW
46	S7			X	wild-type strain	SW
47	S8		X		wild-type strain	SW
48	S11		X		wild-type strain	SW
49	S12		X		wild-type strain	SW
50	S14		X		wild-type strain	SW
51	S16		X		wild-type strain	SW
52	IV22	X			wild-type strain	Italy (IT)
53	IV33	X			wild-type strain	IT
54	IV40		X		wild-type strain	IT
55	IV42		X		wild-type strain	IT
56	IV49	X			wild-type strain	IT
57	IV50	X			wild-type strain	IT
58	IV52			X	wild-type strain	IT
59	IV68		X		wild-type strain	IT
60	IV69		X		wild-type strain	IT
61	IV72			X	wild-type strain	IT
62	IV75		X		wild-type strain	IT
63	IV125		X		wild-type strain	IT
64	IV127		X		wild-type strain	IT
65	IV145	X			wild-type strain	IT
66	IV148			X	wild-type strain	IT

Table S2. TFBSTools hits found in the *kuz* intron region where *FBti0019170* is inserted.

PWM matrix ID	Start position	End position	TFBStools score	Location	Sequence
PB0148.1	13,565,189	13,565,202	0.9532646	inside TE	AAATAAAAAAAAAA
PB0148.1	13,565,664	13,565,677	0.9556583	outside TE	TTTTTTTATTATTT
PB0148.1	13,565,675	13,565,688	0.9532646	outside TE	TTTTTTTTTTATTT
PB0148.1	13,605,418	13,605,431	0.9599062	outside TE	GTGTTTCTAATAAA

Supplementary Figure S1. Results of the LD_{50} performed to determine the appropriate concentration of $ZnCl_2$ to perform the phenotypic assays. In (A) columns represent the average of surviving flies per tube (20 flies). Surviving flies were counted for five days after the first adults emerged. In (B) column represents the average of embryo to adult viability. In each column, error bars represent the standard error of the mean (50 embryos).

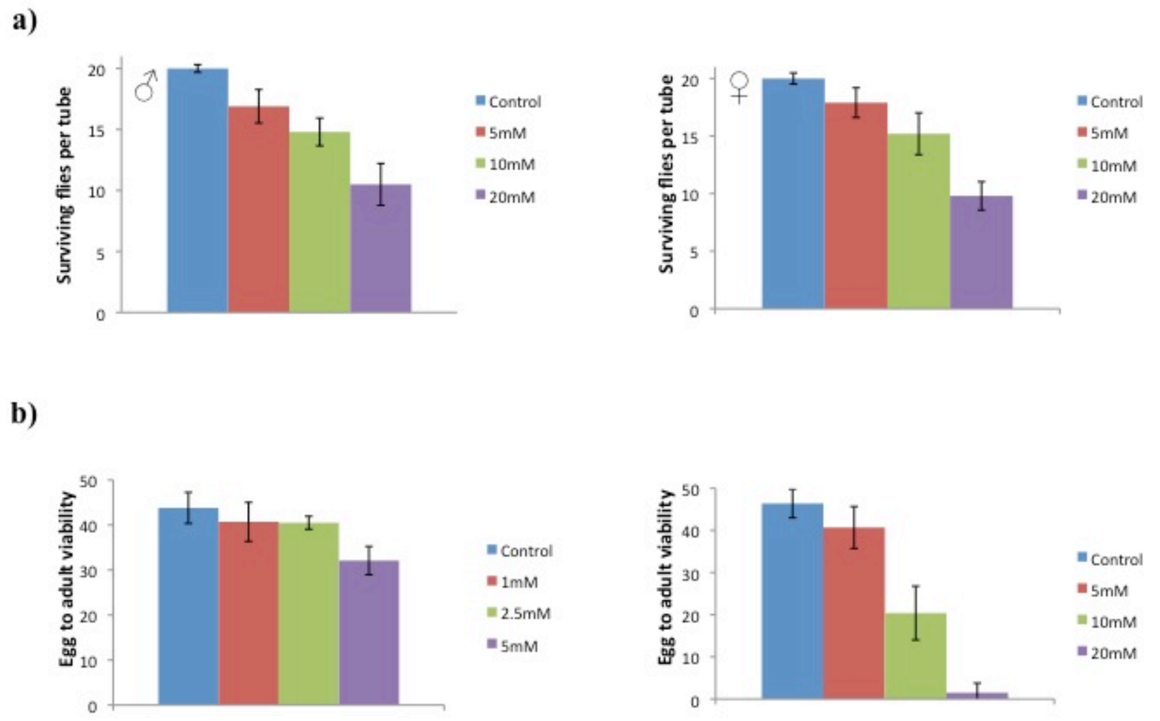

Supplementary Figure S2. *Kuz-overexpressing* flies show a higher level of *kuz* expression

Three replicates of 20 five-day-old female flies each were incubated at 25C for 48 hours before checking the level of expression of *kuz*. RNA extraction and qRT-PCR was performed as described in Methods. *kuz-overexpressing* flies show a higher level of expression compared with *kuz-wildtype* flies (t-test p-value = 0.03)

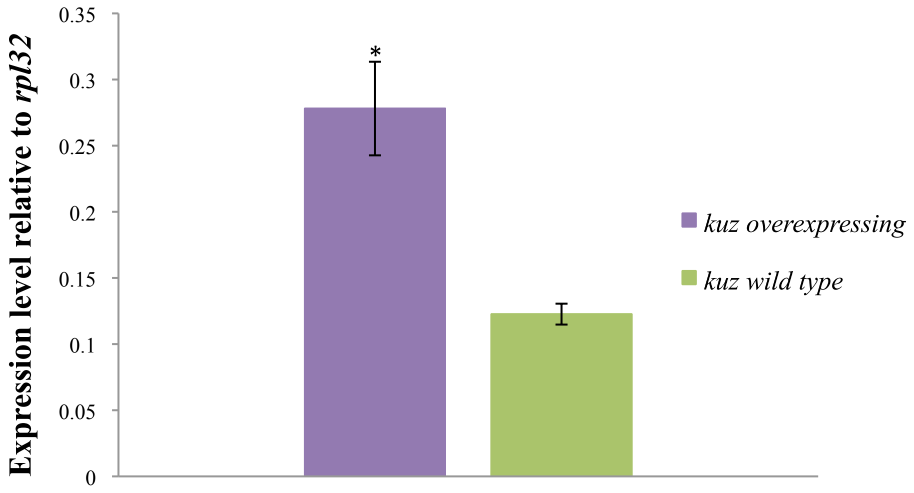

Supplementary Figure S3. Normalized expression ratio of *Act5C* and *rpl32* in nonstress *vs* zinc stress conditions.

Five day-old outbred flies (30 females and 50 males) were separated by sex and transferred to standard fly food as well as food containing 20 mM zinc for 48 hours before freezing them in liquid nitrogen. We did three biological replicas for each sex and condition. RNA extraction was performed as described in Methods. No differences in gene expression level in stress vs nonstress conditions were found (t-test p-value > 0.05)

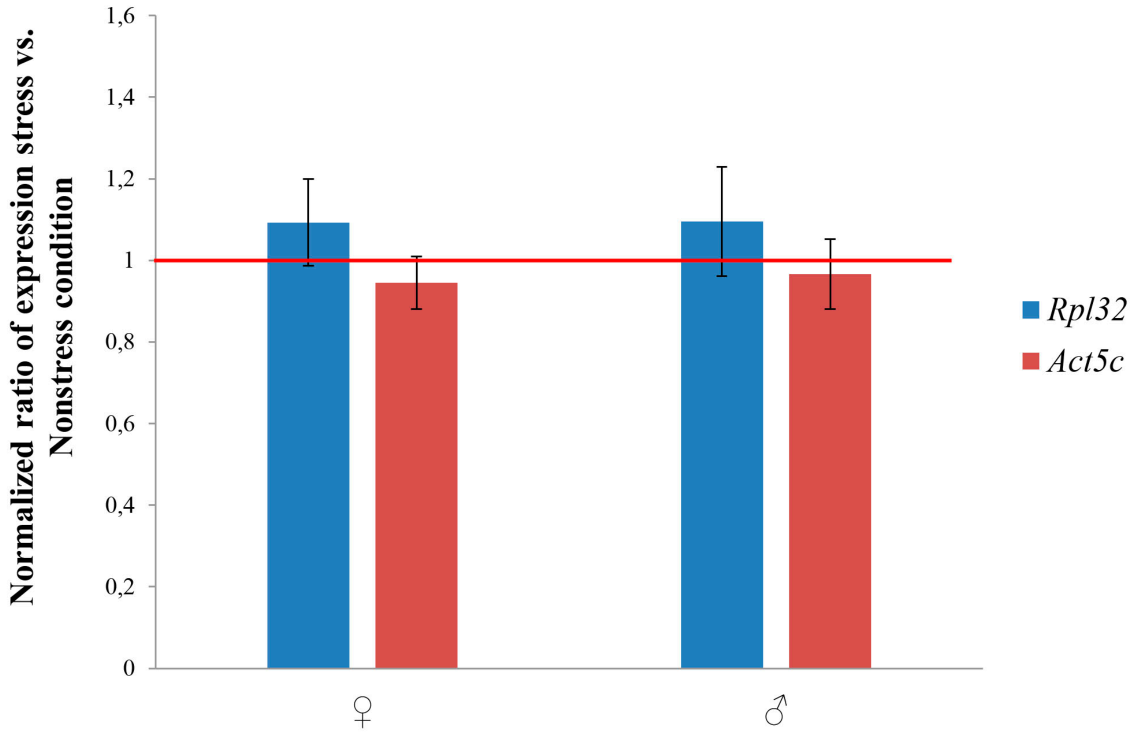

Supplement: Supporting Information [file srep42663-s1.pdf]
